# Successful interdisciplinary management of the misdeployment of two self-expanding stents into the internal carotid artery: a case report

**DOI:** 10.1186/1752-1947-4-397

**Published:** 2010-12-09

**Authors:** Dominik Jost, Helfried Meissner, Henning von Loewensprung, Thomas Guethe, Thomas Hupp, Hans Henkes

**Affiliations:** 1 Department of Vascular Surgery, Klinikum Stuttgart, Stuttgart, Germany; 2 Department of Anaesthesiology and Intensive Care, Klinikum Stuttgart, Stuttgart, Germany; 3 Department of Cardiovascular Disease, Klinikum Stuttgart, Stuttgart, Germany; 4 Faculty of Medicine University of Tuebingen, Tuebingen, Germany; 5 Department of Neuroradiology, Klinikum Stuttgart, Stuttgart, Germany; 6 Faculty of Medicine University of Duisburg-Essen, Essen, Germany

## Abstract

**Introduction:**

With the widespread use of carotid artery stenting, previously unknown technical mistakes of this treatment modality are now being encountered. There are multiple strategies for the treatment of in-stent restenosis. With regard to surgical management, endarterectomy and patch plasty are favored. To the best of our knowledge, this report is the first description of a complete stent removal by the eversion technique.

**Case presentation:**

We report the case of a 63-year-old Caucasian man with misdeployment of two stents into his stenotic proximal internal carotid artery, resulting in a high-grade mechanical obstruction of the internal carotid artery lumen. With the contralateral internal carotid artery already occluded and associated stenoses of both proximal and distal vertebral arteries, an interdisciplinary therapeutic concept was applied. Bilateral balloon angioplasty and stenting of the proximal and distal stenotic vertebral arteries were carried out to provide sufficient posterior collateral blood flow, followed by successful surgical stentectomy and carotid endarterectomy using the eversion technique. Duplex scanning and neurological assessments were normal over a 12-month follow-up period.

**Conclusions:**

Interdisciplinary treatment is a recommended option to protect patients from further impairment. Further evaluation in larger studies is highly recommended.

## Introduction

Stroke is the most common cause of disability. Prevention of stroke by carotid endarterectomy (CEA) or carotid artery stenting (CAS) is widely accepted, and they are basically equivalent treatment modalities [1,2]. The endovascular treatment of internal carotid stenoses is an appropriate treatment method not just for patients at high surgical risk. It is not unexpected that procedural safety and complication rates of CAS are closely related to the operator's skill and the institutional experience with the technique. This can be expressed in terms of caseload or patient enrollment numbers into clinical trials [3]. With the widespread and unregulated use of CAS, however, complication rates could increase and their management is sometimes a challenge for vascular specialists. Apart from the inherent risks of stenting procedures (for example, stent thrombosis, distal emboli, hyperperfusion, hemorrhage and so on), a variety of technical failures have also been observed. They include, among others, sizing issues with overdilation or underdilatation, distal wire injury, and disconnection of protection filters, with their respective clinical sequelae. Here, we report the case of a patient in whom a proximal internal carotid artery (ICA) stenosis was stented in another hospital. At presentation to our institution, the apparent misdeployment of the two stents in a partly overlapping position was found ('hugging' stents). Well co-ordinated endovascular and surgical management saved our patient from further impairment.

## Case presentation

**Figure 1:**
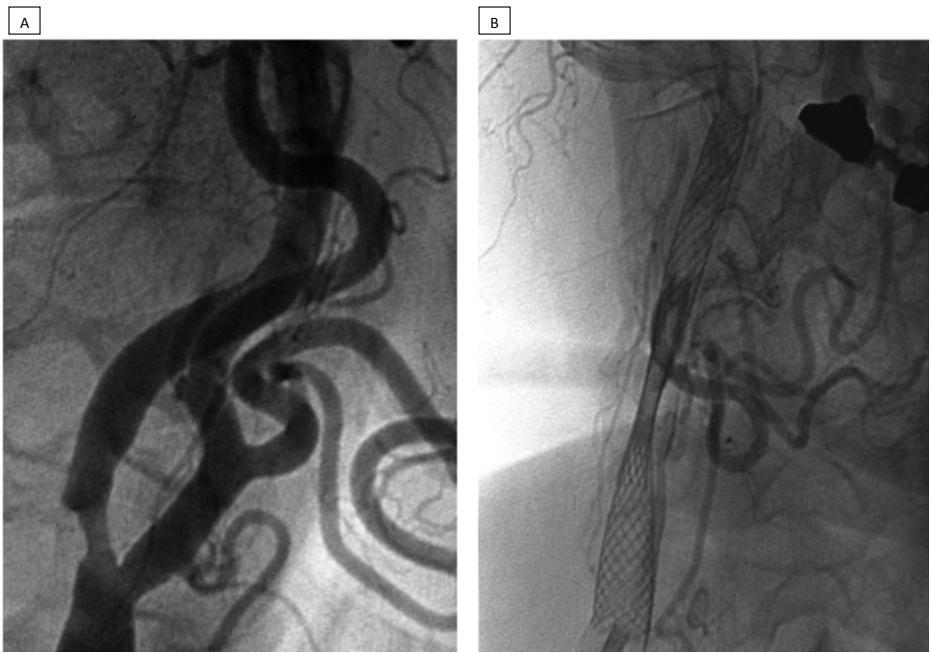
**A) Digital subtracted angiography (DSA) of the right common carotid artery reveals a mid-grade proximal internal carotid artery stenosis**. B) In another institution two Wallstents were deployed without balloon angioplasty, resulting in a significant residual stenosis of the ICA. Due to monoplane imaging in apparently only one projection, the incondite position of the stents remained unrecognized.

A 63-year-old Caucasian man initially presented with an asymptomatic 55% stenosis of the right proximal ICA (Figure 1A) to another institution. The left ICA was found to be occluded; the vessels of the posterior circulation were not examined. Our patient underwent an endovascular procedure at the other institution, which included the deployment of two 7 mm/40 mm Wallstents (Wallstent, Boston Scientific Corporation, Natick, MA, USA) (Figure 1B). The reason why two stents were inserted without balloon angioplasty remains unexplained. Then, three months later, our patient was referred to our hospital with clinical signs and symptoms of a transient left hemispheric ischemia, including global aphasia, right hemiparesis, and paresthesia of the right upper extremity. His cardiovascular risk factors included arterial hypertension, hyperlipidemia and non-insulin dependent diabetes mellitus. Our patient was a non-smoker. Our patient's history included severe coronary heart disease, cardiac insufficiency (New York Heart Association stage II) and recurrent atrial fibrillation, which altogether resulted in an American Society of Anesthesiologists (ASA) physical status of IV.

**Figure 2:**
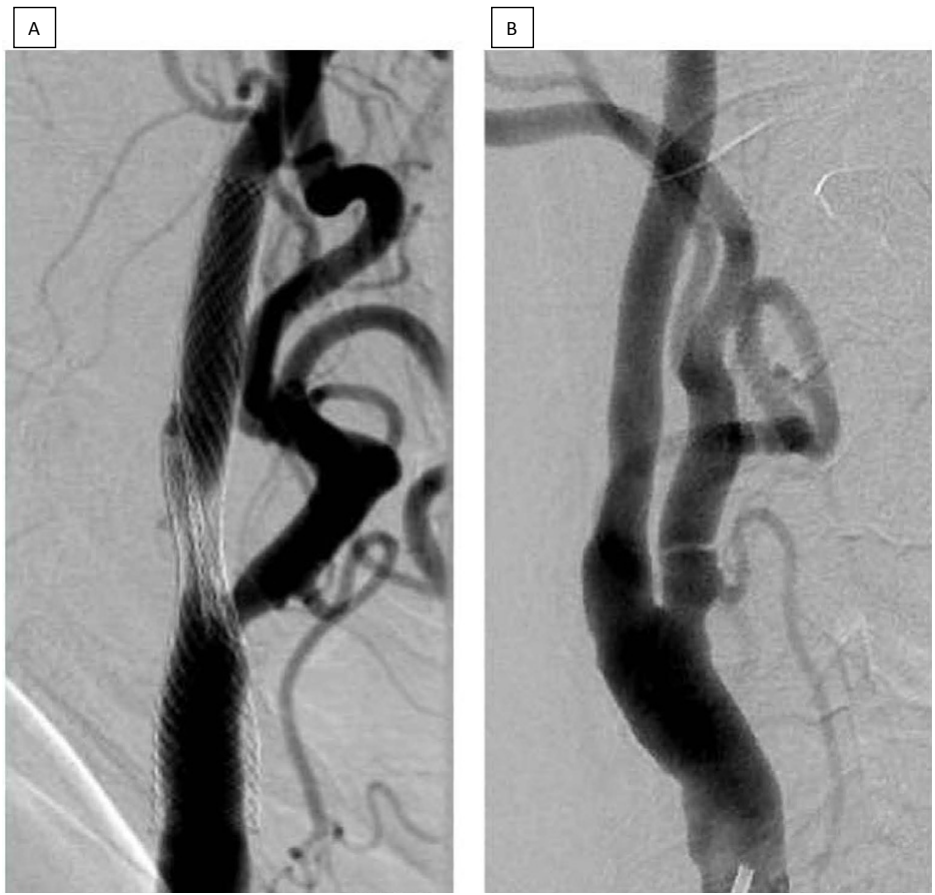
**A) During the diagnostic investigation for transient left hemispheric ischemic signs and symptoms, misdeployment of the two stents became apparent**. Instead of being inserted in a coaxial way, the distal end of the lower and the proximal end of the upper stent were found side by side. B) Both stents and the proximal internal carotid artery stenosis were removed surgically with excellent reconstruction of the proximal carotid artery lumen after six months.

Magnetic resonance imaging and angiography including diffusion-weighted imaging did not reveal any ischemic lesions of the left hemisphere. Digital subtracted angiography (DSA) of the supra-aortic and intra-cranial vessels confirmed the occlusion of the left ICA and revealed a highly effective mechanical obstruction of the right ICA caused by two stents deployed in an overlapping side-by-side position (Figure 2A). Further atherosclerotic lesions included significant stenoses of both proximal vertebral arteries (V1), a stenosis of the entire left V4 segment and a focal stenosis at the junction of the right V4 segment with the basilar artery. The anterior communicating artery and the right posterior communicating artery were widely patent and the left external carotid artery contributed to the supply of the left hemisphere via the ophthalmic artery. When giving his informed consent, our patient was informed of a peri-procedural and post-procedural mortality and morbidity rate of between 3% and 5%, and an increased risk of peri-operative bleeding due to the antiplatelet medication. Our patient accepted this, and the off-label use of both the Coroflex (Coroflex Please, B Braun Melsungen AG, Melsungen, Germany) and Enterprise (Codman Enterprise, Raynham, MA, USA) stents.

The first step of the treatment strategy focused on the posterior circulation stenoses in order to improve the potential collateral supply during subsequent stentectomy and CEA. Dual platelet antiaggregation with acetylsalicylic acid and clopidogrel was initiated. Under general anesthesia the stenoses of both vertebral artery origins were treated with short drug-eluting stents (Coroflex), followed by the stent percutaneous transluminal angioplasty of the left and right V4 stenosis using a combination of moderately undersized balloon dilatation and deployment of oversized self-expanding stents (Enterprise).

The surgical stent removal from the right ICA combined with CEA completed the treatment. Stentectomy and CEA were carried out under regional anesthesia.

As routinely, we operated using ultrasound-guided regional anesthesia (MicroMaxx, Sonosite GmbH, Erlangen, Germany) of the cervical plexus using 20 cc of lidocaine 1% (Xylocain, Braun Melsungen AG, Melsungen, Germany) and 50 cc of ropivacaine 0.375% (Naropin, Astra Zeneca GmbH, Wedel, Germany). The dispensation of analgosedation allowed our patient to be awake throughout the operation, while neurological function was monitored by assessing the level of consciousness and our patient's motor function on the left side. At first the proximal ICA was dissected circumferentially beyond the level of the carotid bifurcation after uneventful clamping. The removal of the proximal stent from the common carotid artery was then possible without any neurological deficit, and we decided to proceed with the eversion technique. The simultaneous removal of the two 'hugging' stents together with the atherosclerotic plaque in the proximal ICA was possible. The ICA and common carotid artery (CCA) were reanastomosed using a 6/0 polypropylene suture in a continuous fashion. Intra-operative angiographic assessment was performed to ensure patency and in order to control the distal end of the plaque removal. Our patient made an uneventful recovery with no additional neurological deficit.

Histological examination revealed a thickened layer of arterial neointima. Duplex scanning was within normal limits after five days and three months, and was confirmed by DSA after six months (Figure 2B). Follow-up duplex scanning surveillance and neurological assessments were unremarkable after 12 months.

## Discussion

With experienced staff at dedicated centers, CEA and CAS are considered equally safe and efficient methods for the treatment of proximal carotid artery stenoses. However, carotid artery angiography and stenting requires proper training [4]. In institutions with a sufficiently large caseload, low complication rates of CAS can be achieved [5]. However, the technical risks and the clinical sequelae of CAS procedures performed by inexperienced operators have also been noted [6].

Our case report deserves some discussion. Whether an asymptomatic mid-grade ICA stenoses with contralateral ICA occlusion should be treated is the subject of an ongoing debate [7,8]. The deployment of a self-expanding stent without balloon angioplasty has been proposed by others [9], but would certainly not be our preferred technique. The deployment of two stents is hard to justify, and in this particular case is likely just a technical mistake. Unfortunately, the reason why two stents were inserted in this way remains unexplained by the operator at the other hospital. The non-coaxial deployment, which apparently remained unrecognized by the operator, made the situation even worse. The significant residual stenosis (Figure 1B) can be interpreted as a failure to improve the cerebral perfusion and should have prompted immediate action. The omission of the examination of the posterior circulation vessels, which would have shown the complexity of the cerebral blood supply, is also an area for criticism.

Our subsequent efforts applied generally accepted endovascular and surgical methods, and started with the deployment of a short drug-eluting stent into the proximal vertebral artery stenoses [10]. For treatment of the basilar artery stenosis, a combination of undersized balloon dilatation followed by the deployment of a moderately oversized self-expanding stent was used [11]. The following surgical stentectomy and CEA was not carried out in a standard fashion.

At present, surgical experience with complications after stenting is limited [12]. In the case of our patient, with a symptomatic occlusion of the left ICA and a high-grade mechanical obstruction of the right proximal ICA, we 'prepared' him for the operative stentectomy with temporary clamping of the right ICA by improving the collateral supply using endovascular means. With regard to the anesthesiological and surgical methods, local anesthesia during CEA with the eversion technique offers the possibility of continuous neurological assessment; an inherent advantage over general anesthesia. The eversion endarterectomy enabled the simultaneous removal of the two stents and the underlying carotid plaque. As the proximal part of the two 'hugging' stents could be removed easily, we decided not to use the standard endarterectomy with patch plasty. Thus, a biological carotid reconstruction without extraneous tissue was possible in this re-do procedure. To the best of our knowledge this is the first description of a stentectomy by carotid endarterectomy with the eversion technique [13]. A single suture for reanastomosis of ICA and CCA after plaque eversion reduces the risk of bleeding under high antiplatelet medication with acetylsalicylic acid and clopidogrel in comparison to the standard patch plasty.

The strategy of removal of the two 'hugging' stents with prior hemodynamic 'preparation' led to a good clinical outcome for our patient, without any further neurological deficit during follow-up of 12 months.

## Conclusion

Post-procedural complication management in vascular medicine is a continuous task requiring interdisciplinary co-operation. The increasing numbers of stent procedures will increase the related surgical expertise. Recommendations for re-do procedures required by local complications of carotid stenting are: (1) the earlier the better, (2) biological reconstruction with the eversion technique is beneficial, and (3) institutions which offer the full range of therapeutic options on site are advantageous.

Further evaluation in larger studies is highly recommended.

## Consent

Written informed consent was obtained from the patient for publication of this case report and any accompanying images. A copy of the written consent is available for review by the Editor-in-Chief of this journal.

## Competing interests

The authors declare that they have no competing interests.

## Authors' contributions

DJ, HvL and TH performed the surgical procedure and drafted the case report. TG and HH performed the endovascular procedure. HM participated in the diagnostic and therapeutic decisions and was responsible for follow-up examinations. TH and HH made major contributions to writing the manuscript. All authors read and approved the final manuscript.
